# Clinical Criteria to Guide Antineuronal Antibody Testing for People With Early and Persistent Psychosis Attending Mental Health Services

**DOI:** 10.1016/j.bpsgos.2025.100612

**Published:** 2025-09-17

**Authors:** Gemma McKeon, Andrea Baker, Stefan Blum, David Gillis, Kerri Prain, Judith M. Greer, George Bruxner, Michael E. Benros, Sean Hatherill, Shuichi Suetani, Dan Siskind, Kate Murphy, Hitesh Joshi, Jackie Curtis, Brian O’Donoghue, Iain Macmillan, Eva Malacova, Anita Pelecanos, Patrick Waters, Belinda Lennox, Nicola Warren, James G. Scott

**Affiliations:** a Queensland Centre for Mental Health Research, The Park Centre for Mental Health, Brisbane, Queensland, Australia; b West Moreton Health Psychology, West Moreton Health, Ipswich, Queensland, Australia; c Child Health Research Centre, The University of Queensland, Brisbane, Queensland, Australia; d Department of Neurosciences, Princess Alexandra Hospital, Brisbane, Queensland, Australia; e Pathology Queensland, Royal Brisbane and Women’s Hospital, Brisbane, Queensland, Australia; f University of Queensland Centre for Clinical Research, Royal Brisbane and Women’s Hospital, Brisbane, Queensland, Australia; g Caboolture Hospital, Caboolture, Queensland, Australia; h Copenhagen Research Centre for Biological and Precision Psychiatry, Mental Health Centre Copenhagen, Copenhagen University Hospital, Copenhagen, Denmark; i Department of Clinical Medicine, Faculty of Health and Medical Sciences, University of Copenhagen, Copenhagen, Denmark; j Metro South Addiction and Mental Health Service, Brisbane, Queensland, Australia; k School of Medicine and Dentistry, Griffith University, Brisbane, Queensland, Australia; l Faculty of Health, Medicine, and Behavioural Sciences, The University of Queensland, Brisbane, Queensland, Australia; m Metro North Mental Health, Brisbane, Queensland, Australia; n Faculty of Medicine, University of New South Wales, Kensington, New South Wales, Australia; o Eastern Suburbs Mental Health Service, South Eastern Sydney Local Health District, Sydney, New South Wales, Australia; p School of Medicine, University College Dublin, Dublin, Ireland; q Department of Psychiatry, St Vincent’s University Hospital, Dublin, Ireland; r Alfred Hospital, Melbourne, Victoria, Australia; s Headspace Early Psychosis, Frankston, Victoria, Australia; t Monash University, Melbourne, Victoria, Australia; u QIMR Berghofer Medical Research Institute, Brisbane, Queensland, Australia; v Nuffield Department of Clinical Neurosciences, University of Oxford, Oxford, United Kingdom; w Department of Psychiatry, University of Oxford, Oxford, United Kingdom; x Child and Youth Mental Health Service, Children’s Health Queensland, Brisbane, Queensland, Australia

**Keywords:** Autoimmune encephalitis, Autoimmune psychosis, Early psychosis, NMDA receptor, Schizophrenia, Screening

## Abstract

**Background:**

Early detection of autoimmune psychosis (AP) mediated by antineuronal antibodies (Abs) is critical for achieving optimal clinical outcomes. However, evidence remains limited regarding who should be tested and how Ab-positive cases should be managed. In this large-scale study, we evaluated proposed clinical criteria for targeted Ab testing in psychiatric services and described the clinical course of seropositive patients.

**Methods:**

Individuals with early psychosis (EP) or persistent psychosis (PP) were prospectively assessed with clinical criteria to determine high- or low-risk status for AP. Blood samples were collected for Ab testing using a fixed cell-based assay. Seropositive individuals were invited for detailed review, including clinical, functional, and cognitive assessments at baseline and a 12-month follow-up. Blood samples were collected from 754 individuals (EP: *n* = 352, PP: *n* = 402).

**Results:**

Abs were present in 2.3% (17/754), including 3.4% (12/352) of patients with EP and 1.2% (5/402) of patients with PP. AP was confirmed in 2 cerebrospinal fluid (CSF)–positive high-risk individuals (total: 2/754, 0.3%; EP: 1/352, 0.3%; PP: 1/402, 0.2%). Both improved with immunotherapy. Although some low-risk patients were seropositive, none were diagnosed clinically with AP.

**Conclusions:**

AP prevalence was low in this cohort. Targeted testing informed by clinical high-risk criteria successfully identified 2 immunotherapy-responsive AP cases. This approach appears feasible but requires further validation. People with psychosis and high-risk AP features should be considered for Ab testing in sera and CSF where indicated. Further research is required to embed targeted Ab testing into mental health services.

## Plain Language Summary

In this study, we explored whether certain clinical signs can help mental health services to identify a rare autoimmune form of psychosis caused by brain antibodies. We tested 754 people for these antibodies and found 2 cases of autoimmune psychosis who were classified as high risk. They responded to immunotherapy. Using high-risk clinical criteria to guide antibody testing decisions holds promise, but further research is needed before this approach can be adopted as standard practice.

Autoimmune encephalitis (AE), most commonly mediated by immunoglobulin G antineuronal antibodies (Abs) targeting the NMDA receptor (NMDAR), often presents with acute psychosis, usually preceding neurological and autonomic dysfunction (1,2). Nonpsychiatric features may be subtle or absent initially (1,3). From this, the concept of autoimmune psychosis (AP) has emerged, describing psychiatric syndromes suspected to be caused by Abs disrupting neuronal targets (4). Detecting AP cases is challenging. Significant cognitive and behavioral disturbance occurs in psychosis irrespective of the cause (1,5).

Interest in AP has increased across psychiatry, neurology, and immunology, driven by the promising role of immunotherapy for psychosis. However, the boundaries differentiating AP, AE, and non–immune-mediated psychoses are debated (6,7), with presentations ranging from unequivocal AE with psychosis to cases where an autoimmune contribution is suspected but not easily confirmed (8). Awareness of AP has been transformative for psychiatric research and practice. Australian and New Zealand Clinical Practice Guidelines recommend universal Ab screening in first-episode psychosis (FEP) (9) to expedite AP identification and treatment; however, increased Ab surveillance in mental health services has introduced unanticipated challenges (10,11).

AP diagnosis is based on clinical assessment and usually requires cerebrospinal fluid (CSF) analysis indicative of central nervous system (CNS) involvement, which can be challenging in individuals with psychosis (4,12). Serum Ab testing is often used initially due to minimal invasiveness; however, results often warrant further investigation depending on the clinical presentation (1,11). NMDAR Ab seroprevalence in psychosis is estimated at 0.73%, higher in first-episode (2.18%) than multi-episode (0.16%) samples, with live cell-based assay (CBA) being more sensitive than commercially available fixed CBAs (13). There are limitations to the diagnostic precision of commercially available assays used for serum testing, with potential for both false positives and false negatives (14,15). Some individuals with AP lack overt neurological signs (3,16), and reliance on serum testing alone may delay detection and immunotherapy due to false-negative results. Conversely, in the absence of clear AE clinical features, seropositivity can create uncertainty about immunotherapy (10,11) and prompt unnecessary investigations and treatment in cases that are ultimately false positives (17).

Improving AP diagnostic accuracy and patient outcomes (18, 19, 20) requires stronger collaboration between psychiatry and neurology (21,22) and clearer methods for identifying psychosis cases requiring serum Ab testing, CSF investigations, and neurological consultation. Routine lumbar puncture at first episode does not increase case detection (23), and seropositivity alone predicts AE diagnosis poorly (24). The clinical presentation should take precedence over serum Ab testing results in determining the need for CSF investigation. Universal Ab screening has low pretest probability, supporting the development of more targeted strategies (10,11,24,25).

The Graus criteria were designed to streamline AE diagnosis (26), but precision is diminished in cases in which psychiatric symptoms predominate (27). To direct testing toward individuals at heightened risk and increase detection probability, various groups (11,23,28,29) have summarized AP warning signs. These incorporate neuroimaging, electroencephalography, and CSF testing, which may hinder real-world utility. Consensus guidelines for possible, probable, and definite AP (4) face similar constraints, leading to misdiagnosis (23,29). Categorical criteria do not capture the complexity of psychiatric presentations or accommodate delays in paraclinical investigations. Tools designed to highlight the likelihood of immune involvement without establishing diagnosis offer a promising complement to categorical frameworks (30).

Based on illness course and observable or directly reportable characteristic AP symptoms (5,31), we propose concise high-risk clinical criteria (Table 1) independent of paraclinical investigations and applicable in patients with early psychosis (EP) and persistent psychosis (PP) (11). These criteria are aimed at distinguishing patients more likely to have syndromes associated with known pathogenic Abs and guide serum Ab testing and CSF analysis decisions. Incidentally identified inflammatory markers (32) may require follow-up but are not the focus of the criteria. The criteria are not diagnostic for AP, which incorporates clinical features, antibody status, and other investigations (4). In a retrospective study (33), these criteria demonstrated similar validity to the widely accepted “flags” of Herken and Prüss (28).

**Table 1:** Clinical Antineuronal Antibody Testing Criteria to Categorize High Risk and Low Risk of Autoimmune Psychosis

Criteria	Early Psychosis	Persistent Psychosis
High Risk	Criteria 1 and one or more of criteria 2–7 are met.	Any criteria below are met.
Low Risk	High risk criteria are not met.	None of the criteria below are met.
1	Rapid onset (<1 month) of the FEP	Persistent positive symptoms that are refractory to antipsychotic therapy from illness onset, i.e., not a relapsing-remitting illness
2	New-onset neurological signs, e.g., seizures, abnormal involuntary movements, and EPS such as orolinguofacial dyskinesias, limb/trunk choreoathetosis, oculogyric crisis, dystonia, and rigidity	History of neurological signs, e.g., seizures, abnormal involuntary movements, and EPS such as orolinguofacial dyskinesias, limb/trunk choreoathetosis, oculogyric crisis, dystonia, and rigidity, which are probably unrelated to medication
3	New-onset severe cognitive or language involvement, e.g., inattention, memory impairment, disorganization, inability to comprehend speech, reduced verbal expression, or mutism	Persistent, severe cognitive or language involvement, e.g., inattention, memory impairment, disorganization, inability to comprehend speech, reduced verbal expression, or mutism, refractory to antipsychotic therapy
4	Catatonia or intermittent agitation or excitatory states	History of catatonia
5	Atypical, heightened sensitivity to EPSE of antipsychotic medication, e.g., dystonias, Parkinsonism, akathisia	History of atypical, heightened sensitivity to the EPSE of antipsychotic medication, e.g., dystonias, Parkinsonism, akathisia
6	Illness severity necessitates management with ECT	History of management with ECT due to illness severity
7	Fluctuating mental state consistent with delirium-type presentation	NA

ECT, electroconvulsive therapy; EPS, extrapyramidal symptom; EPSE, extrapyramidal side effect; FEP, first episode of psychosis.

In this study, we aimed to prospectively validate the high-risk clinical criteria in EP and PP cohorts with the goal of increasing the probability of identifying AP. Additional aims were to 1) describe a case series of seropositive patients with psychosis and 2) compare fixed and live CBAs. We hypothesized that Abs would be present in both risk groups, but AP would only occur in high-risk cases.

## Methods and Materials

### Recruitment

The Metro South Hospital and Health Service Human Research Ethics Committee approved this study (HREC/2019/QMS/52220). Fourteen sites were involved, including inpatient units and community teams across 3 Australian states. Mental health teams were informed about the study and asked to refer eligible patients. The study was embedded within clinical services, and consequently, the proportion of eligible patients offered screening is unknown. Screening and recruitment commenced in July 2020 and concluded in August 2022.

### Participants

Eligibility criteria included 1) ages 14 to 60 years, 2) clinical diagnosis of any psychotic disorder, 3) no history of immunotherapy for AP, 4) ability to communicate in English and follow study procedures, and 5) absence of physical illness that would prevent study completion. Because Ab testing is part of routine care of people with psychosis in Australia, a consent waiver was obtained for screening patients. Ab-seropositive cases were eligible for further study phases (see the Supplement, section S1a) and approached to provide informed consent. Full physical assessment, including CSF analysis, was recommended for Ab-seronegative high-risk individuals who remained treatment refractory. No initially seronegative Ab cases were referred. Parents or guardians provided consent together with eligible patients of ages 14 to 17 years.

### Procedures

During phase 1, clinicians explained the rationale for investigating Abs to patients with psychosis. Using the clinical criteria (Table 1), assenting participants were categorized by clinicians as high risk or low risk of AP prior to Ab testing. Additional information collected included date of birth, sex, clinical diagnosis, estimated symptom duration, and illness severity rated using the Clinical Global Impressions (CGI) scale (34). Duration of psychosis was used to categorize the EP (≤1 year) and PP (>1 year) groups.

During phases 2, 3, and 4, the following information was collected from eligible consenting participants and medical records: 1) demographics; 2) medical/psychiatric history, including symptoms, investigations, treatments, and response; and 3) measures of symptom severity, functioning, and neurocognitive ability (see Supplement, section S1a).

Ten milliliters of blood was analyzed for Abs by a specialist Australian diagnostic neuroimmunology laboratory using commercial fixed CBAs (for full details, see Supplement, section S1b). To further assess antibody status and provide an assay comparison, sera from a subsample of consenting patients were sent to the neuroimmunology laboratory at John Radcliffe Hospital in Oxford, United Kingdom, for analysis using a live CBA, scored with fluorescence microscopy. This included samples testing positive through fixed CBA and a selection of negative sera. No samples were tested for voltage-gated potassium channel (VGKC) antibodies using live CBA, which directly evaluated clinically relevant antibodies targeting leucine-rich glioma-inactivated 1 (LGI1) and contactin-associated protein-like 2 (CASPR-2). CSF samples were not analyzed with live CBA.

AP diagnosis was determined through multidisciplinary specialist consensus, involving expertise from neurology, immunology, psychiatry, and neuropsychiatry and informed by guidelines for AE (26) and AP (4). This was based on review of all available clinical and paraclinical results, including symptomatology, paraclinical investigations, serum and CSF analyses, and treatment response.

### Statistical Analyses

Descriptive statistics were used to summarize group characteristics and Ab testing results. The frequency with which different targeted testing criteria were applied was tabulated and summarized descriptively. Group differences were examined using independent *t* tests and χ^2^ tests for continuous and categorical variables, respectively. Fisher’s exact test was applied as indicated. Statistical analyses were conducted using IBM SPSS Statistics, version 30 (35). Statistical significance was set at *p* < .05.

## Results

### Participants

**Figure 1:**
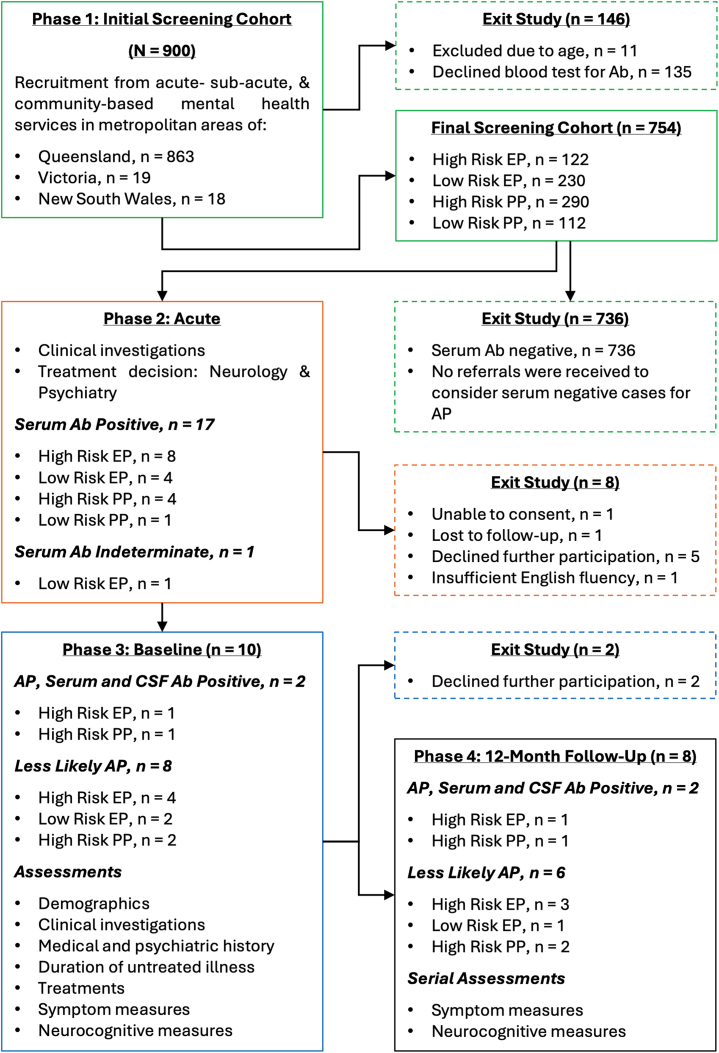
Participant recruitment and assessments. Ab, antineuronal antibody; AP, autoimmune psychosis; CSF, cerebrospinal fluid; EP, early psychosis; PP, persistent psychosis.

Recruitment is summarized in Figure 1. Of 900 referred patients, 146 were excluded due to refusal of serological testing (*n* = 135, 15%) or age (*n* = 11, 1.2%). Complete information was obtained from 132 patients who refused serological testing. There were no significant differences in sex (χ^2^_1,__*n*__= 889_ = 2.29, *p* = .153) or age (*t*_886_ = −1.38, *p* = .168) based on blood test refusal status (see Supplement, section S2a). Those who completed blood tests presented with higher symptom acuity than those who refused (*t*_884_ = 6.39, *p* < .001). Participants with EP completed testing more often than participants in the PP cohort (χ^2^_3,__*n*__= 888_ = 44.97, *p* < .001) (Supplement, section S2a).

The final EP and PP cohorts with complete data comprised 352 and 402 patients, respectively. Participant characteristics and Ab testing results are summarized by psychosis duration (EP vs. PP) and risk group (high risk vs. low risk) in Table 2. Sex did not differ significantly between the EP and PP cohorts (χ^2^_1,__*n*__= 754_ = 2.62, *p* = .105) but did vary by risk group (χ^2^_3,__*n*__= 754_ = 8.57, *p* < .03) (Supplement, section S2b), likely reflecting a higher-than-expected proportion of female high-risk participants with EP (53%). Half of the EP sample was assigned the nonspecific diagnosis of first episode of psychosis, whereas schizophrenia was the most common diagnosis (66%) in the PP group. Compared with the PP group, participants with EP were younger (*t*_752_ = −12.86, *p* < .001) (Supplement, section S2b), with shorter illness duration (by group definition) and higher symptom acuity (*t*_751_ = 6.99, *p* < .001) as assessed by the CGI scale (34).

**Table 2:** Early and Persistent Psychosis, High- and Low-Risk Group Characteristics, and Antineuronal Antibody Testing Results

	Early Psychosis			Persistent Psychosis		
	HR, *n* = 122	LR, *n* = 230	Total, *N* = 352	HR, *n* = 290	LR, *n* = 112	Total, *N* = 402
Demographics						
Age, years	27.7 (11.9)	29.0 (10.4)	28.5 (10.9)	39.7 (10.7)	36.8 (11.5)	38.9 (11.0)
Female	65 (53.3%)	93 (40.4%)	158 (44.9%)	110 (37.9%)	47 (42.0%)	157 (39.1%)
Male	57 (46.7%)	137 (59.6%)	194 (55.1%)	180 (62.1%)	65 (58.0%)	245 (60.9%)
Symptom Severity, CGI^a^	5.7 (1.0)	5.2 (1.1)	5.4 (1.1)	4.9 (1.4)	4.1 (1.9)	4.7 (1.7)
Symptom Duration Estimate	27.1 days [1 day–1 year]	3.5 months [2 days–6 years]	2.7 months [1 day–6 years]	14.6 years [1–44 years]	9.0 years [1–44 years]	13 years [1–44 years]
Clinical Diagnosis, Provisional						
First episode of psychosis	68 (55.7%)	110 (47.8%)	178 (50.6%)	4 (1.4%)	6 (5.4%)	10 (2.5%)
Delusional disorder	0 (0.0%)	0 (0.0%)	0 (0.0%)	2 (0.7%)	1 (0.9%)	3 (0.7%)
Brief psychotic disorder	2 (1.6%)	4 (1.7%)	6 (1.7%)	1 (0.3%)	1 (0.9%)	2 (0.5%)
Schizophreniform disorder	1 (0.8%)	6 (2.6%)	7 (2.0%)	1 (0.3%)	2 (1.8%)	3 (0.7%)
Schizophrenia	0 (0.0%)	9 (3.9%)	9 (2.6%)	206 (71.0%)	58 (51.8%)	264 (65.7%)
Schizoaffective disorder	0 (0.0%)	1 (0.4%)	1 (0.3%)	43 (14.8%)	12 (10.7%)	55 (13.7%)
Substance-induced psychotic disorder	3 (2.5%)	15 (6.5%)	18 (5.1%)	6 (2.1%)	4 (3.6%)	10 (2.5%)
Psychotic disorder due to medical condition	1 (0.8%)	1 (0.4%)	2 (0.6%)	1 (0.3%)	0 (0.0%)	1 (0.2%)
Unspecified psychosis	29 (23.8%)	63 (27.4%)	92 (26.1%)	17 (5.9%)	21 (18.8%)	38 (9.5%)
BD/mania, psychotic features	15 (12.3%)	17 (7.4%)	32 (9.1%)	8 (2.8%)	6 (5.4%)	14 (3.5%)
MDD/MDE, psychotic features	3 (2.5%)	4 (1.7%)	7 (2.0%)	1 (0.3%)	1 (0.9%)	2 (0.5%)
Ab, F-CBA						
Positive total^b^	8 (6.6%)	4 (1.7%)	12 (3.4%)	4 (1.4%)	1 (0.9%)	5 (1.2%)
NMDAR	6 (4.9%)	–	6 (1.7%)	3 (1.0%)	1 (0.9%)	4 (1.0%)
VGKC, negative LGI1, CASPR-2	1 (0.8%)	1 (0.4%)	2 (0.6%)	–	–	–
CASPR-2	1 (0.8%)	1 (0.4%)	2 (0.6%)	–	–	–
GFAP	–	–	–	1 (0.3%)	–	1 (0.2%)
GAD	–	2 (0.9%)	2 (0.6%)	–	–	–
Negative	114 (93.4%)	225 (97.8%)	339 (96.3%)	286 (98.6%)	111 (99.1%)	397 (98.8%)
Indeterminate	–	1 (0.4%)	1 (0.3%)	–	–	–
NMDAR Ab in CSF^c^	1 (0.8%)	–	1 (0.3%)	1 (0.3%)	–	1 (0.2%)

Values are presented as mean (SD), mean [range], or *n* (%).

Ab, antineuronal antibody; BD, bipolar disorder; CASPR-2, contactin-associated protein-like 2; CGI, Clinical Global Impressions; CSF, cerebrospinal fluid; EP, early psychosis; F-CBA, fixed cell-based assay; GAD, glutamic-acid decarboxylase; HR, high risk; LGI1, leucine-rich glioma inactivated 1; LR, low risk; MDD, major depressive disorder; MDE, major depressive episode; NMDAR, NMDA receptor; PP, persistent psychosis; VGKC, voltage-gated potassium channel.

a Rated by clinicians who referred patients for Ab testing and completed the clinical high-risk criteria.

b Serum Ab frequency in combined EP, PP sample: 17/754 (2.3%), 18/754 (2.4%) including indeterminate result.

c CSF NMDAR Ab frequency in combined EP, PP sample: 2/754 (0.3%).

### Antibody-Seropositive Cases

In total, 18 of 754 participants with psychosis (2.3%) were Ab seropositive, including 12 of 352 (3.4%) participants with EP, 5 of 402 participants with PP (1.2%), and 1 low-risk participant with EP who returned an indeterminate result initially assumed to be seropositive (Table 2 and Figure 1). For 8 seropositive cases who did not consent to participate further, treatment decisions and outcomes were unknown. Ten seropositive cases consented to baseline, and 8 were re-assessed 12 months later. Their demographic and clinical characteristics, investigations, treatments, and symptomatic responses are described in Tables 3 (EP) and 4 (PP). The application of clinical testing criteria for consenting participants is summarized in Table 5. Results of symptom, functional, and cognitive measures from baseline and follow-up are available in the Supplement, section S3.

**Table 3:** Summary of Clinical Information From Consenting Seropositive Participants With Early Psychosis

	Case 1, HR	Case 2, HR	Case 3, HR	Case 4, HR	Case 5, HR	Case 6, LR	Case 7, LR
Demographics	Female, 37 years	Female, 22 years	Male, 16 years	Female, 23 years	Male, 17 years	Male, 23 years	Male, 27 years
Diagnosis	Autoimmune psychosis: NMDAR encephalitis	Unspecified non-organic psychosis	Mania, psychotic symptoms	Substance-induced psychosis	Acute schizophrenia-like psychotic disorder	Substance-induced psychosis	Unspecified non-organic psychosis
Symptoms at Testing	Irritability, insomnia, and anxiety precede acute decline, syncopal event, self-harm, confusion, agitation, aggression, hallucinations, catatonia, and autonomic disturbance	Acute amnesia, bizarre behavior, delusions, and thought disorder on background of adverse childhood experiences and chronic hallucinations	Acute decline with new medication: persecutory/grandiose ideas, labile mood, disorganized, pressured speech, tangentiality, and insomnia	Acute onset erratic behavior, agitation, hallucinations, and bizarre delusions on background of recent infection, traumatic event, and substance use	Acute decline with changes to medications for neurodevelopmental diagnoses: aggression, agitation, erratic behavior, delusions, hallucinations, and incoherent speech	Gradual decline; disinhibition, erratic behavior, aggression, insomnia, hallucinations, and delusions	Acute-onset perceptual disturbance, delusions, odd behavior, mild thought disorder, and fatuous affect
Untreated Illness Duration	17 days	14 days	2 months, 4 days	14 days	5 days	5 months, 10 days	20 days
Investigations							
Ab serum, F-CBA/L-CBA	NMDAR, GAD positive/all negative	NMDAR positive/NMDAR low positive	NMDAR positive/NMDAR low positive	VGKC positive (LGI1, CASPR-2 negative)/All negative	NMDAR positive/NMDAR low positive	CASPR-2 positive/CASPR-2 positive	Indeterminate/NMDAR positive
Ab CSF/other CSF	NMDAR positive/elevated WCC	Ab negative/normal	Not collected	Not collected	Not collected	Not collected	Not collected
MRI brain	Normal	Normal	Normal	Not completed	Not completed	Not completed	Not completed
EEG	Abnormal	Not completed	Normal	Not completed	Abnormal	Normal	Not completed
Treatments							
Psychiatric	Olanzapine	Olanzapine	Olanzapine, sodium valproate	Aripiprazole	Lithium, risperidone	Aripiprazole, olanzapine, venlafaxine	Aripiprazole
Immunotherapy	IVMp (2 courses), IVIg (2 courses), rituximab, maintenance IVIg	No	No	No	No	No	No
Response^a^							
Acute	Poor	Responded	Responded	Responded	Responded	Responded	Responded
12 months	Remission	Relapsing remitting	Remission	Remission	Remission	Persistent symptoms	Remission

Ab, antineuronal antibody; CASPR-2, contactin-associated protein-like 2; CSF, cerebrospinal fluid; EEG, electroencephalography; F-CBA, fixed cell-based assay; GAD, glutamic-acid decarboxylase; HR, high risk; IVIg, intravenous immunoglobulin; IVMp, intravenous methylprednisolone; L-CBA, live cell-based assay; LGI1, leucine-rich glioma-inactivated 1; LR, low risk; MRI, magnetic resonance imaging; NMDAR, NMDA receptor; VGKC, voltage-gated potassium channel complex.

a Refers to the clinical response documented in medical records to all treatments, assessed during the acute phase and at the 12-month follow-up. For case 1, this relates to immunotherapy.

**Table 4:** Summary of Clinical Information From Consenting Seropositive Participants With Persistent Psychosis

	Case 8, HR	Case 9, HR	Case 10, HR
Demographics	Male, 49 years	Male, 40 years	Male, 31 years
Diagnosis	Autoimmune psychosis (paranoid schizophrenia)	Paranoid schizophrenia	Schizoaffective disorder, manic type
Symptoms at Testing	Chronic treatment-refractory psychotic symptoms: residual thought disorder, paranoia, amotivation, social isolation, and cognitive dysfunction; stable for extended period	Chronic relapsing-remitting psychotic symptoms: disorganization, poor self-care, and isolation; stable for extended period	Chronic relapsing-remitting psychotic/affective symptoms: higher-level cognitive difficulties; stable for extended period
Illness Duration	26 years	15 years	7 years
Investigations			
Ab serum, F-CBA/L-CBA	NMDAR positive/all negative	NMDAR positive/NMDAR positive	GFAP^a^ positive/all negative
Ab CSF/other CSF	NMDAR positive/elevated protein	All negative/normal	Lumbar puncture unsuccessful
MRI brain	Normal	Normal	Normal
EEG	Abnormal	Normal	Not completed
Treatments			
Psychiatric	Clozapine, amisulpride, mirtazapine	Clozapine, venlafaxine, naltrexone	Clozapine, sodium valproate
Immunotherapy	IVIg induction and maintenance	No	No
Response^a^			
Acute	Responded	Stable	Stable
12 months	Sustained improvement	Stable	Stable

Ab, antineuronal antibody; CSF, cerebrospinal fluid; EEG, electroencephalography; F-CBA, fixed cell-based assay; GFAP, glial fibrillary acidic protein; HR, high risk; IVIg, intravenous immunoglobulin; L-CBA, live cell-based assay; MRI, magnetic resonance imaging; NMDAR, NMDA receptor.

a Refers to the clinical response documented in medical records to all treatments, assessed during the acute phase and at the 12-month follow-up. For case 8, this relates to immunotherapy.

**Table 5:** Application of Clinical Antineuronal Antibody Testing Criteria for Autoimmune Psychosis in Consenting Seropositive Cases

Criteria	Case 1	Case 2	Case 3	Case 4	Case 5	Case 6	Case 7	Case 8	Case 9	Case 10
Onset/Duration	Yes	Yes	Yes	Yes	Yes	No	Yes	Yes	No	No
Neurologic Sx	Yes	No	No	No	No	No	No	Yes	No	No
Cognition	Yes	Yes	Yes	Yes	Yes	No	No	Yes	No	No
Catatonia/Agitation	Yes	No	No	No	Yes	No	No	No	No	No
EPSE	No	No	No	No	No	No	No	No	No	No
ECT	No^a^	No	No	No	No	No	No	Yes	Yes	Yes
Delirium	Yes	Yes	No	Yes	No	No	No	–	–	–
Classification	HR EP	HR EP	HR EP	HR EP	HR EP	LR EP	LR EP	HR PP	HR PP	HR PP
Criteria Total	5/7	3/7	2/7	3/7	3/7	0/7	1/7	4/6	1/6	1/6
AP Confirmed?	Yes	No	No	No	No	No	No	Yes	No	No

AP, autoimmune psychosis; ECT, electroconvulsive therapy; EP, early psychosis; EPSE, extrapyramidal side effect; HR, high risk; LR, low risk; PP, persistent psychosis; Sx, signs/symptoms.

a ECT was considered in this case immediately prior to identification of NMDA receptor antibodies.

AP was confirmed in 2 cases with NMDAR Abs in sera and CSF (case 1 EP, Table 3; case 8 PP, Table 4), both prospectively classified as high risk. The clinical presentation, investigations, treatments, and course for these patients are detailed in Box 1. Briefly, case 1 (EP) presented with an acute deterioration with psychiatric and neurological signs and symptoms. Case 8 (PP) had a lengthy history of treatment-resistant schizophrenia and less obvious neurological symptoms, with myoclonic jerks when treated with clozapine. Following immunotherapy, some positive outcomes were observed on cognitive, functional, and symptom measures. Case 1 demonstrated intact cognitive performance and functional status following a life-threatening illness and a lengthy admission. For case 8, clinician-rated symptoms reduced with immunotherapy, while functional disability remained. Seropositive cases lacking confirmatory CNS Ab detection were considered less likely to have AP and did not receive immunotherapy. Four seropositive participants with EP were in remission at 12 months with standard psychiatric care (cases 3, 4, 5, and 7). Two seropositive participants with PP (cases 9 and 10) had no change in their illness over 12 months.

Box 1 **Autoimmune Psychosis Case Histories**

**Table:** 

Case 1 – High-Risk Early Psychosis
A 37-year-old woman with no significant psychiatric or medical history presented with 1 month of anxiety, panic attacks, and insomnia. She was admitted after an episode of altered consciousness and deliberate self-harm, rapidly developing fluctuating catatonia, hallucinations, thought disorder, cognitive impairment, and behavioral disturbance. Oral medications for agitation were minimally effective. She was prescribed regular olanzapine. Brain MRI was unremarkable. Routine blood test results were normal except for mildly elevated white cell count. EEG demonstrated mild encephalopathy. On day 2, neurological examination identified no abnormalities. She became increasingly confused and ceased eating, drinking, and speaking by day 6, and continued hallucinating. She developed tachycardia, hypertension, and enuresis and started holding her breath with oxygen saturation levels decreasing to 60%. Behavioral disturbance worsened. Emergency ECT was considered. NMDAR Abs were identified in serology, prompting further neurological consultation. CSF was aseptic, with elevated white cell count and presence of Abs confirming an NMDAR encephalitis diagnosis. Upon transfer to the intensive care unit, she was sedated, intubated, and treated with intravenous methylprednisolone (1 g daily, 5 days) followed by 5 days of IVIg. Ovarian teratoma was suspected radiologically, with laparoscopic bilateral oophorectomy performed (day 11). No teratoma was found. She developed hospital-acquired infections in the context of immunosuppression, requiring various antibiotics. EEG was abnormal, with high altitude delta wave. Neurological progress was slow. Tracheostomy was required to facilitate ventilator weaning. Rituximab (2 g total) was commenced on day 23. She developed seizures and central movement disorder and was prescribed levetiracetam and valproate. Alertness gradually improved after a second course of IVIg (5 days), and she was transferred to a rehabilitation ward on day 59 when she could speak. Initial progress with multidisciplinary input was positive, with relapse on day 72 requiring transfer back to the neurology ward, risperidone and quetiapine for hallucinations, and another 3-day course of IV methylprednisolone (3 g total). She was discharged on day 102. Cognitively, she performed within normal limits for age at baseline, 134 days after initial presentation, and 12 months later. NMDAR Abs were negative in serum. Performance across most tasks was stable, with verbal fluency improvement and incidental visual memory decline noted. Psychotic symptoms as evaluated by the PANSS increased by 50% from baseline to 12 months but reflected normal or borderline illness ratings. Psychologically, DASS ratings were not suggestive of elevated distress at either time point. There was slight functional impairment at 12 months as measured by the SOFAS score of 78.
Case 8 – High-Risk Persistent Psychosis
A 49-year-old man with a 26-year history of paranoid schizophrenia initially experienced acute-onset mental illness in early adulthood in the context of substance use. Enduring psychiatric symptoms derailed a promising professional career. Over the course of psychiatric management, psychotic symptoms were refractory to all treatments, including 1) amisulpride, 2) aripiprazole, 3) clozapine, 4) fluphenazine, 5) olanzapine, 6) quetiapine, 7) risperidone, 8) stelazine, 9) thioridazine, and 10) zuclopenthixol. Response to all antipsychotics, including clozapine and various combinations, had been partial. Side effects included paradoxical reaction to olanzapine and intolerable myoclonic jerks and enuresis on clozapine, which resolved with cessation. He had been admitted more than 10 times. He presented as chronically thought disordered, with auditory hallucinations, delusional beliefs, blunted affect, amotivation, and social isolation. There had been multiple suicide attempts. He was re-trialed on clozapine and developed myoclonic jerks again but made positive psychiatric improvement. EEG was abnormal at this point, with mild diffuse encephalopathy. He was commenced on sodium valproate after neurological consultation, which addressed involuntary movements. He gained weight and developed impaired glucose tolerance and raised blood lipids, leading to a diabetes mellitus type 2 diagnosis several years later, at which point he was commenced on metformin. Medical history was otherwise unremarkable. Mental state had been stable on clozapine for many years prior to referral for targeted antineuronal Ab testing. He presented with mild paranoia and thought disorder and more prominent negative symptoms. Upon identification of serum NMDAR Abs, neurology referral was made. No abnormalities were detected on clinical examination or brain MRI. EEG indicated mild diffuse encephalopathy, with no epileptiform features. Lumbar puncture confirmed NMDAR Abs in CSF, which had raised protein levels. Following 5-day IVIg induction, he received monthly maintenance IVIg. Cognitively, baseline performance was largely normal for age, with an isolated executive deficit. Scores remained stable at follow-up, with some variability on tests of working memory, flexibility, and theory of mind. Above average premorbid intellect raised suspicion for subtle decline from baseline. Functional impairments persisted. Quality of life qualitatively increased at follow-up. Ratings of depressive, anxious, and stress-related symptoms were normal across both assessments. Clinical response was noted in psychotic symptoms, with PANSS total decreasing by 25% from the mildly/moderately ill to mildly ill range. He remained seropositive for NMDAR Abs at follow-up.

Ab, antineuronal antibody; CSF, cerebrospinal fluid; DASS, Depression Anxiety Stress Scale; ECT, electroconvulsive therapy; EEG, electroencephalography; IVIg, intravenous immunoglobulin; MRI, magnetic resonance imaging; NMDAR, NMDA receptor; PANSS, Positive and Negative Syndrome Scale; SOFAS, Social and Occupational Functioning Assessment Scale.

### Assay Comparison

The 10 sera samples from seropositive consenting participants assessed with fixed CBA were analyzed with live CBA, along with 20 negative samples. Testing outcomes agreed in 80% of cases (24/30) (Table 6). Three samples that were negative according to fixed CBA were low positive for NMDAR (*n* = 2) and LGI1 (*n* = 1) antibodies using live CBA. The latter sample was not tested for antibodies to LGI1/CASPR-2 using fixed CBA, with the discordant outcome being related to the process of testing, not the assays. An indeterminate fixed CBA result (case 7) was NMDAR positive using live CBA. In cases 1 and 8, where CSF Abs were detected using fixed CBA, live CBAs were negative.

**Table 6:** Live and Fixed CBA Testing Comparisons From Consenting Seropositive, Indeterminate, and Selected Seronegative Fixed CBA Subsample

Case ID	Fixed CBA Result	Live CBA Result	Agreement
1	NMDA positive	Negative	No
2	NMDA positive	NMDA low positive	Yes
3	NMDA positive	NMDA low positive	Yes
4	VGKC positive (LGI1, CASPR-2 negative)	Negative	Partial yes—noting discordance in stepwise procedure
5	NMDA positive	NMDA low positive	Yes
6	CASPR-2 positive	CASPR-2 positive	Yes
7	Indeterminate	NMDA positive	No
8	NMDA positive	Negative	No
9	NMDA positive	NMDA positive	Yes
10	GFAP positive	Negative	Partial yes—noting that GFAP was not assessed via live CBA
11	Negative	Negative	Yes
12	Negative	Negative	Yes
13	Negative	Negative	Yes
14	Negative	Negative	Yes
15	Negative	Negative	Yes
16	Negative	Negative	Yes
17	Negative	Negative	Yes
18	Negative	NMDA low positive	No
19	Negative	Negative	Yes
20	Negative	Negative	Yes
21	Negative	Negative	Yes
22	Negative	NMDA low positive	No
23	Negative	Negative	Yes
24	Negative	Negative	Yes
25	Negative	Negative	Yes
26	Negative	Negative	Yes
27	Negative	Negative	Yes
28	Negative	Negative	Yes
29	Negative	Negative	Yes
30	Negative	LGI1 low positive	No

Total concordance was 80% agreement: 24 agree (yes/partial yes) and 6 disagree (no).

CASPR-2, contactin-associated protein-like 2; CBA, cell-based assay; GFAP, glial fibrillary acidic protein; LGI1, leucine-rich glioma-inactivated 1; VGKC, voltage-gated potassium channel.

### Targeted Testing Criteria Clinical Application

Statistical validation of the clinical criteria was not possible due to the small number of confirmed AP cases (*n* = 2). A significant relationship was found between Ab blood test results and risk group classification (Fisher’s exact test, *p* < .021), with small effect size (Cramer’s V = 0.123, *p* < .009) (see the Supplement, section S2c). The highest proportion of positive test results was identified in the high-risk EP group (8/122; 6.6%). Table 5 displays the assessment of seropositive cases against high-risk clinical criteria. The 2 participants with AP (cases 1 and 8) met most high-risk criteria. All NMDAR-seropositive patients with EP demonstrated rapid onset and severe cognitive or language involvement. Seropositive participants with PP had all been treated with electroconvulsive therapy due to illness severity.

### EP Targeted Testing

Eight of 12 seropositive cases with EP were classified as high risk. As noted, one case of AP due to NMDAR antibodies was confirmed in the high-risk EP cohort (case 1). Glutamic-acid decarboxylase (GAD) antibodies were moderately elevated (titer 139 IU/mL), likely reflecting background autoimmunity. In another 5 instances of NMDAR antibody-seropositive cases, 1 was CSF negative (case 2), 2 responded to psychiatric care (cases 3 and 5), and another 2 did not consent to participate. Case 4 was VGKC seropositive, negative for LGI1 or CASPR-2 antibodies, and responded to aripiprazole. AP was excluded based on symptom course, response to psychiatric treatments, and the absence of antibodies to clinically relevant targets. AP could not be excluded in 1 CASPR-2–positive high-risk EP case who declined further tests or participation.

Of the 4 seropositive low-risk patients with EP, 2 who were GAD positive were lost to follow-up or declined investigations. Titers were elevated (>2000 IU/mL), and in 1 case, high IA2 (40 IU/mL) suggested autoimmune diabetes. Their diabetic status was unknown, and further assessment was not possible to establish clinical relevance. The third case returned a VGKC-positive LGI1/CASPR-2–negative result of unlikely clinically relevance and was not eligible for further participation. Case 6 was CASPR-2 positive, with input from neurology sought and lumbar puncture completed without detection of CSF antibodies. Comorbid substance abuse, psychosocial complexity, and engagement issues complicated assessment and management. Another low-risk participant (case 7) returned an indeterminate result, which was negative 3 weeks later. Case 7 did not participate at the 12-month follow-up. Medical records indicated ongoing stability with standard psychiatric treatment.

### PP Targeted Testing

Four of the 5 seropositive cases with PP were classified as high risk, including 3 cases with NMDAR antibodies. One case of NMDAR antibody–mediated AP was identified and improved with immunotherapy (case 8). One case was Ab negative in CSF (case 9). Another declined further participation. One individual was GFAP seropositive, but lumbar puncture was unsuccessful (case 10). One low-risk PP NMDAR seropositive case did not consent to further tests or participation.

## Discussion

In this cohort of 754 individuals receiving clinical care for psychosis, 18 (2.3%) were seropositive via fixed CBA, and 2 (0.3%) were confirmed to have AP based on clinical presentation, investigations, and immunotherapy response. As expected, serological Abs were detected across all risk groups, with AP confirmation limited to high-risk participants. Serological prevalence of Abs by risk group was, in descending order, as follows: high-risk EP: 6.6%, low-risk EP: 1.7%, high-risk PP: 1.4%, and low-risk PP: 0.9%. While the frequency of seropositive results was associated with risk classification, the effect size was small, and the clinical relevance of serum Abs was uncertain in most cases. It remains possible that confinement of confirmed AP cases to the high-risk groups occurred by chance.

Within the high-risk groups, 1 participant with EP (1/122, 0.82%) and 1 individual with PP (1/290, 0.35%) were confirmed as having AP. This suggests that AP is rare, even in individuals with high-risk features. Case 1 had a prototypical presentation of NMDAR encephalitis with acute psychosis and life-threatening autonomic dysfunction requiring intensive care, similar to early case descriptions (36). The second participant (case 8) with immune-mediated psychosis had been diagnosed with treatment-refractory schizophrenia for 26 years. Importantly, both were classified prospectively as high risk of AP and met the majority of the clinical testing criteria.

The AP cases in this study raise questions about the boundaries between AP, AE, and mental illness, a topic of debate (4,6,7). The shared immunological pathogenesis, but markedly different symptom profiles and trajectories mirror the variable onset and course observed in other CNS autoimmune disorders, such as multiple sclerosis (37) and neuropsychiatric lupus (38). Case 1 met criteria for both definite NMDAR encephalitis (26) and AP (4). Case 8 had subtle neurological symptoms and a lengthy history of treatment-refractory schizophrenia, limiting application of categorical guidelines. Emerging frameworks, such as synaptic and neuronal autoantibody–associated psychiatric syndromes, may conceptualize such cases better (6). Further research is needed to understand distinct symptom pathways. Despite incomplete diagnostic concordance, CNS NMDAR antibodies and immunotherapy response strongly supported an autoimmune process in case 8. This scenario demonstrates the value of structured tools to assess the likelihood of AP. The Neuropsychiatric Checklist for Autoimmune Psychosis (30) and our briefer high-risk clinical criteria offer practical methods for highlighting individuals warranting Ab investigations.

**Figure 2:**
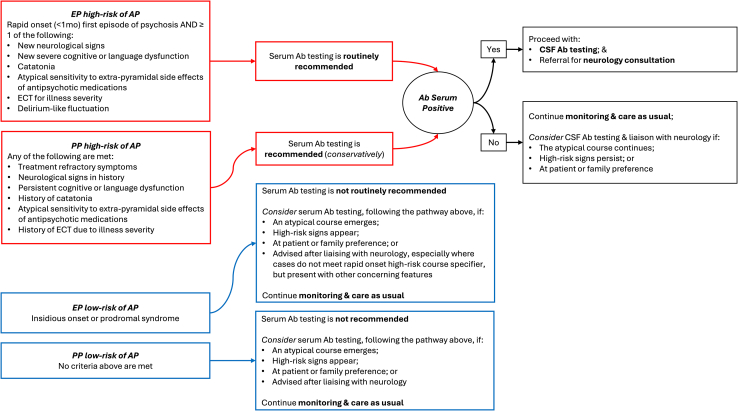
Decision tree. Ab, antineuronal antibody; AP, autoimmune psychosis; CSF, cerebrospinal fluid; ECT, electroconvulsive therapy; EP, early psychosis; PP, persistent psychosis.

These results support the proposed clinical criteria for prioritizing Ab testing for individuals at high risk of AP in mental health services. Figure 2 suggests pathways for serum and CSF Ab testing and neurology consultation based on clinical features in EP and PP. Investigations should be undertaken with awareness that most seropositive cases are likely false positives for an immune-mediated illness. However, delayed AP detection can result in a chronic, disabling illness, and this must be considered as a differential diagnosis for individuals with psychosis and high-risk features. In PP, conservative Ab testing is recommended but may be justified in treatment-refractory high-risk cases, in keeping with meta-analytic evidence regarding Ab prevalence and illness stage (13). Case 8 demonstrates that prolonged psychopathology without substantial neurological deterioration can occur. Immunotherapy resulted in a measurable reduction in psychotic symptoms. We have previously reported immunotherapy benefits for other individuals for whom AP diagnosis was delayed (20).

The discordant live and fixed CBAs were noteworthy, consistent with reports of inconsistencies between methods (39) and emphasizing the importance of a formulation that integrates symptom course, AP risk factors, investigation results, and input from multiple specialties. Irrespective of method, serum Ab results neither confirm nor exclude AP and should not be interpreted in isolation. Several Ab-negative findings via fixed CBA were seropositive on live CBA, warranting further investigation. These patients did not consent to further participation, and their outcomes were unknown. Given that 14% to 28% of CSF-positive individuals lack detectable serum Abs (14,15), where diagnostic uncertainty persists and the patient remains severely ill despite psychiatric care, CSF Ab testing may be required, and neurological consultation should be obtained. No serum-negative participants were referred for AP consideration, potentially missing false negatives. Further education and training could address this gap in clinical knowledge.

The range of Abs linked to neuropsychiatric dysfunction is growing (1,40). It is likely that as-yet uncharacterized Abs exist, contributing to psychosis without accompanying neuroinflammatory syndromes (41). The Abs tested for in routine care and in this study were associated with AE, but autoantibodies can cause pathology via other mechanisms without inducing CNS inflammation (42). The clinical significance of seropositive results remains uncertain for people with psychosis without evident encephalitis or CNS involvement, where psychiatric care facilitates improvement as observed in cases 3 and 5. These scenarios may reflect transient immunological contributions to symptom onset that remit spontaneously, as seen in some inflammatory neuropathies (43). They may represent a prognostically different presentation along an AP spectrum, analogous to the evolving understanding of multiple sclerosis, which has shifted from rigid subtypes to a disease activity continuum (44). No seropositive individual with Ab-negative or untested CSF received immunotherapy, limiting contribution to ongoing debate regarding evidence-based clinical pathways and the relevance of serological Abs in these cases (7,21).

In this study, we confirmed fewer-than-anticipated AP cases. We cannot rule out that additional cases were overlooked. Our previous prospective study conducted in the same geographic area found that 5 of 113 (4.4%) patients with FEP had an immune-mediated illness (45). The current study was undertaken during the COVID-19 pandemic. Perhaps social distancing contributed to reduced immunological triggers circulating in the population. It is also possible that AP is now more widely recognized, with emergency department presentations being referred to neurology, not psychiatry. This would provide further justification against universal EP screening. Closer study of the adoption and implications of heightened Ab monitoring in people with psychosis is warranted.

### Methodological Considerations

To our knowledge, this is the second practical application of an Ab testing strategy for people with psychosis, informed by clinical risk criteria. In a recent study, 78 patients with FEP were referred for Ab testing based on indicators of higher or lower pretest probability (29). Two cases of NMDAR encephalitis were identified; however, only people with warning signs were tested, leaving it unclear how many people without these signs might have had Abs. We applied our clinical criteria on a broader scope, encompassing more patients across multiple sites and extending beyond EP to include PP. This approach holds promise and can be safely implemented on a large scale. Our study demonstrates the real-world challenges that clinicians face in deciding which psychiatric patients should undergo Ab testing and the subsequent actions based on test results.

For pragmatic reasons aligned with naturalistic settings, no structured tool was used to diagnose psychotic disorders. Considering the clinical impressions provided, it was evident that cohorts reflected samples primarily characterized by psychosis, inclusive of diverse psychopathology typical of patients in public mental health services. For similar reasons, risk classifications were known when determining AP diagnoses. Decisions were based on objective data reviewed by an expert multidisciplinary team and guided by established criteria (4,26). However, the potential introduction of bias should be acknowledged.

Blood tests were declined or not possible in 15% of our original sample, with a higher refusal rate among individuals with PP. This undermines the feasibility of universal serum Ab screening in psychosis, and differential participation limits generalizability to the broader PP population. Lumbar puncture was also not feasible for all seropositive individuals. Consequently, there is a risk that some cases of AP went undiagnosed.

False negative results in the seronegative group is another area of uncertainty, especially in high-risk cases. Considering the live CBA subsample, some fixed CBA results were likely false negatives. Our methodology lacked procedures to enable participant contact following Oxford testing, limiting opportunities for follow-up. This is particularly relevant for antibodies against VGKC complex antigens, including LGI1 and CASPR-2, in light of evidence that VGKC antibodies alone are unlikely to hold clinical significance (46). Stepwise assessment of VGKC before LGI1 and CASPR-2 likely missed some seropositive cases. Additionally, only fixed CBA was used for CSF analysis. Regardless of assay type, CSF Ab testing is accepted as precise, with high interpretive clarity (15,39). Systematic assay comparison was limited, but it is unlikely that CSF interpretation was impacted.

There were instances of possible misclassifications within the EP or PP categories. Erroneous recording of illness duration and application of the high-risk clinical criteria likely reflected the complexity of patients and the demands on clinicians providing care for people living with complex mental illness. This highlights the need for criteria to inform Ab testing that are easily applied by busy clinicians working with severely unwell patients.

Statistical validation of the high-risk criteria for predicting AP, including optimal thresholds for recommending Ab testing, was not possible due to smaller confirmed case numbers than anticipated. COVID-19–related disruptions reduced recruitment below the target of 1200 (500 high-risk, 100 low-risk EP, PP). This limited analyses and introduced enrollment bias because referrals prioritized high-risk cases. Larger postpandemic studies are needed to validate and refine the criteria, which proved feasible and clinically informative in this study.

### Conclusions

AP is rare but must not be overlooked. Mental health services should adopt targeted, not universal, Ab testing strategies for psychosis. Patients with EP need Ab testing if illness onset is rapid with severe language or cognitive impairment, and other high-risk features are present. Patients with PP should be tested when clinical criteria strongly indicate AP risk. Further research is needed to enable practice transitions from ad hoc Ab screening to targeted testing of people at high risk of AP.

## Supplementary Material

Supplemental Section S1–S3

Key Resources Table

## References

[1] Dalmau J., Armangué T., Planagumà J., Radosevic M., Mannara F., Leypoldt F. (2019). An update on anti-NMDA receptor encephalitis for neurologists and psychiatrists: Mechanisms and models. Lancet Neurol.

[2] Titulaer M.J., McCracken L., Gabilondo I., Armangué T., Glaser C., Iizuka T. (2013). Treatment and prognostic factors for long-term outcome in patients with anti-NMDA receptor encephalitis: An observational cohort study. Lancet Neurol.

[3] Kayser M.S., Titulaer M.J., Gresa-Arribas N., Dalmau J. (2013). Frequency and characteristics of isolated psychiatric episodes in anti-N-methyl-d-aspartate receptor encephalitis. JAMA Neurol.

[4] Pollak T.A., Lennox B.R., Müller S., Benros M.E., Prüss H., Tebartz van Elst L. (2020). Autoimmune psychosis: An international consensus on an approach to the diagnosis and management of psychosis of suspected autoimmune origin. Lancet Psychiatry.

[5] Warren N., Siskind D., O’Gorman C. (2018). Refining the psychiatric syndrome of anti-N-methyl-d-aspartate receptor encephalitis. Acta Psychiatr Scand.

[6] Al-Diwani A., Pollak T.A., Langford A.E., Lennox B.R. (2017). Synaptic and neuronal autoantibody-associated psychiatric syndromes: Controversies and hypotheses. Front Psychiatry.

[7] Graus F., Dalmau J. (2021). Autoimmune encephalitis or autoimmune psychosis?. Eur Neuropsychopharmacol.

[8] Pollak T.A., Al-Diwani A.A.J., Lennox B. (2017). Neuronal surface autoantibodies, encephalitis and psychosis: from neurology to psychiatry. Adv Clin Neurosci Rehabil.

[9] Galletly C., Castle D., Dark F., Humberstone V., Jablensky A., Killackey E. (2016). Royal Australian and New Zealand College of Psychiatrists clinical practice guidelines for the management of schizophrenia and related disorders. Aust N Z J Psychiatry.

[10] Cohn S.L., Mohan A., Lappin J.M., Curtis J., Scott J.G. (2023). Anti-N-methyl-D-aspartate receptor antibody testing in first-episode psychosis: Universal or targeted testing. J Neuropsychiatry Clin Neurosci.

[11] Scott J.G., Gillis D., Swayne A., Blum S. (2018). Testing for antibodies to N-methyl-D-aspartate receptor and other neuronal cell surface antigens in patients with early psychosis. Aust N Z J Psychiatry.

[12] Abboud H., Probasco J.C., Irani S., Ances B., Benavides D.R., Bradshaw M. (2021). Autoimmune encephalitis: Proposed best practice recommendations for diagnosis and acute management. J Neurol Neurosurg Psychiatry.

[13] Cullen A.E., Palmer-Cooper E.C., Hardwick M., Vaggers S., Crowley H., Pollak T.A., Lennox B.R. (2021). Influence of methodological and patient factors on serum NMDAR IgG antibody detection in psychotic disorders: A meta-analysis of cross-sectional and case-control studies. Lancet Psychiatry.

[14] Bien C.G., Bien C.I., Dogan Onugoren M., De Simoni D., Eigler V., Haensch C.A. (2020). Routine diagnostics for neural antibodies, clinical correlates, treatment and functional outcome. J Neurol.

[15] Gresa-Arribas N., Titulaer M.J., Torrents A., Aguilar E., McCracken L., Leypoldt F. (2014). Antibody titres at diagnosis and during follow-up of anti-NMDA receptor encephalitis: A retrospective study. Lancet Neurol.

[16] Colijn M.A., Ismail Z. (2019). Clinically relevant anti-neuronal cell surface antibodies in schizophrenia spectrum disorders. Neuropsychobiology.

[17] Ketheesan S., Bertram G., Adam R., Stark A., Scott J.G. (2021). Muddying the waters? A false positive case of autoimmune psychosis. Australas Psychiatry.

[18] Charlson F.J., Ferrari A.J., Santomauro D.F., Diminic S., Stockings E., Scott J.G. (2018). Global epidemiology and burden of schizophrenia: Findings from the global burden of disease Study 2016. Schizophr Bull.

[19] McKeon G., Parker S., Warren N., Scott J.G. (2021). The patient experience of recovery following anti-NMDA receptor encephalitis: A qualitative content analysis. J Neuropsychiatry Clin Neurosci.

[20] McKeon G.L., Scott J.G., Spooner D.M., Ryan A.E., Blum S., Gillis D. (2016). Cognitive and social functioning deficits after anti-N-methyl-D-aspartate receptor encephalitis: An exploratory case series. J Int Neuropsychol Soc.

[21] Lennox B.R. (2022). Challenging the psychiatry-neurology divide: The case of autoimmune encephalitis. Nat Rev Neurol.

[22] Pollak T.A., Lennox B.R. (2018). Time for a change of practice: The real-world value of testing for neuronal autoantibodies in acute first-episode psychosis. BJPsych Open.

[23] Guasp M., Giné-Servén E., Maudes E., Rosa-Justicia M., Martínez-Hernández E., Boix-Quintana E. (2021). Clinical, neuroimmunologic, and CSF investigations in first episode psychosis. Neurology.

[24] Ariño H., Coutinho E., Pollak T.A., Stewart R. (2021). Real-world experience of assessing antibodies against the N-methyl-D-aspartate receptor (NMDAR-IgG) in psychiatric patients. A retrospective single-centre study. Brain Behav Immun.

[25] Warren N., McKeon G., Scott J.G. (2024). Replacing universal anti-neuronal antibody screening with clinical assessment and testing of high probability cases in psychotic disorders. Aust N Z J Psychiatry.

[26] Graus F., Titulaer M.J., Balu R., Benseler S., Bien C.G., Cellucci T. (2016). A clinical approach to diagnosis of autoimmune encephalitis. Lancet Neurol.

[27] Warren N., O’Gorman C., Blum S., Kisely S., Swayne A., Flavell J., Siskind D. (2020). Evaluation of the proposed anti-N-methyl-d-aspartate receptor encephalitis clinical diagnostic criteria in psychiatric patients. Acta Psychiatr Scand.

[28] Herken J., Prüss H. (2017). Red flags: Clinical signs for identifying autoimmune encephalitis in psychiatric patients. Front Psychiatry.

[29] Pavăl D., Gherghel-Pavăl N., Căpățînă O.O., Stan A., Raduly L., Budişan L., Micluția I.V. (2024). Neural antibodies in first-episode psychosis patients with warning signs for autoimmune encephalitis. Clin Psychopharmacol Neurosci.

[30] Tebartz van Elst L., Runge K., Meyer P.T., Urbach H., Venhoff N., Prüss H. (2025). The neuropsychiatric checklist for autoimmune psychosis: A narrative review. Biol Psychiatry.

[31] Al-Diwani A., Handel A., Townsend L., Pollak T., Leite M.I., Harrison P.J. (2019). The psychopathology of NMDAR-antibody encephalitis in adults: A systematic review and phenotypic analysis of individual patient data. Lancet Psychiatry.

[32] Warren N., O’Gorman C., Horgan I., Weeratunga M., Halstead S., Moussiopoulou J. (2024). Inflammatory cerebrospinal fluid markers in schizophrenia spectrum disorders: A systematic review and meta-analysis of 69 studies with 5710 participants. Schizophr Res.

[33] Warren N., Flavell J., O’Gorman C., Swayne A., Blum S., Kisely S., Siskind D. (2020). Screening for anti-NMDAR encephalitis in psychiatry. J Psychiatr Res.

[34] Guy W. (1976). ECDEU Assessment Manual for Psychopharmacology—Revised. Rockville, MD: US Department of Health Education, and Welfare, Public Health Service, Alcohol, Drug Abuse and Mental Health Administration.

[35] IBM Corporation (2024). IBM SPSS Statistics for Windows, version 30.0.0.0 (172). 30.0.0.0 (172) ed. https://www.ibm.com/support/pages/downloading-ibm-spss-statistics-30.

[36] Dalmau J., Gleichman A.J., Hughes E.G., Rossi J.E., Peng X., Lai M. (2008). Anti-NMDA-receptor encephalitis: Case series and analysis of the effects of antibodies. Lancet Neurol.

[37] Tullman M.J. (2013). Overview of the epidemiology, diagnosis, and disease progression associated with multiple sclerosis. Am J Manag Care.

[38] Schwartz N., Stock A.D., Putterman C. (2019). Neuropsychiatric lupus: New mechanistic insights and future treatment directions. Nat Rev Rheumatol.

[39] Thouin A., Gastaldi M., Woodhall M., Jacobson L., Vincent A. (2021). Comparison of N-methyl-D-aspartate receptor antibody assays using live or fixed substrates. J Neurol.

[40] Dalmau J., Geis C., Graus F. (2017). Autoantibodies to synaptic receptors and neuronal cell surface proteins in autoimmune diseases of the central nervous system. Physiol Rev.

[41] Ryan A.E., Mowry B.J., Kesby J.P., Scott J.G., Greer J.M. (2019). Is there a role for antibodies targeting muscarinic acetylcholine receptors in the pathogenesis of schizophrenia?. Aust N Z J Psychiatry.

[42] Ludwig R.J., Vanhoorelbeke K., Leypoldt F., Kaya Z., Bieber K., McLachlan S.M. (2017). Mechanisms of autoantibody-induced pathology. Front Immunol.

[43] Hardy T.A., Blum S., McCombe P.A., Reddel S.W. (2011). Guillain-Barre syndrome: Modern theories of etiology. Curr Allergy Asthma Rep.

[44] Lublin F.D., Reingold S.C., Cohen J.A., Cutter G.R., Sørensen P.S., Thompson A.J. (2014). Defining the clinical course of multiple sclerosis: The 2013 revisions. Neurology.

[45] Scott J.G., Gillis D., Ryan A.E., Hargovan H., Gundarpi N., McKeon G. (2018). The prevalence and treatment outcomes of antineuronal antibody-positive patients admitted with first episode of psychosis. BJPsych Open.

[46] van Sonderen A., Schreurs M.W.J., de Bruijn M.A.A.M., Boukhrissi S., Nagtzaam M.M.P., Hulsenboom E.S.P. (2016). The relevance of VGKC positivity in the absence of LGI1 and Caspr2 antibodies. Neurology.

