# Supplementary material for: Clinical Criteria to Guide Antineuronal Antibody Testing for People With Early and Persistent Psychosis Attending Mental Health Services
Source: Biol Psychiatry Glob Open Sci. 2025 Sep 17;6(1):100612. doi: 10.1016/j.bpsgos.2025.100612 (PMC12670449; doi:10.1016/j.bpsgos.2025.100612)
Supplement: Supplemental Section S1–S3 [file mmc1.pdf]

SUPPLEMENTARY INFORMATION

Clinical Criteria to Guide Anti-Neuronal Antibody Testing for People With Early and Persistent Psychosis Attending Mental Health Services

McKeon *et al.*

Supplementary Material 1

S1a: Procedures for Study Phase Two (Acute), Three (Baseline) and Four (12-Months).

Following screening of patients with high-risk clinical criteria and blood collection (phase 1), those who were seropositive for anti-neuronal Ab and those who were anti-neuronal Ab negative but fulfilled high-risk clinical anti-neuronal Ab criteria and were unresponsive to psychiatric treatment were eligible to continue into phase 2 of the study. Individuals who were anti-neuronal Ab positive were contacted by the research team regarding further participation after verbal assent from their treating mental health team. Research team members from the disciplines of psychiatry, neurology, immunology, and neuroimmunology liaised with treating mental health teams in relation to management decisions. Routine clinical care continued. Clinicians from mental health services were encouraged to refer higher risk seronegative patients who were treatment refractory for full physical assessment including lumbar puncture and CSF analysis for anti-neuronal Ab. However, no referrals were received by the research team to approach patients for study consent who were initially seronegative and later found to be CSF anti-neuronal Ab positive.

Baseline assessments were scheduled when participants were well enough to complete the battery of assessments. Assessments were repeated at 12-month follow-up with consenting patients. Participants were reimbursed with gift cards at both points. Information collected from patients and their medical records included: (1) demographics; (2) medical and psychiatric history, including response to treatments; (3) results of routine clinical investigations; and (4) symptom and functional measures (Positive and Negative Syndrome Scale [PANSS; 1], 21-item Depression Anxiety Stress Scale [DASS-21; 2], Modified Rankin Scale [mRS; 3]], Social and Occupational Functioning Assessment Scale [SOFAS; 4], Assessment of Quality of Life [AQoL; 5]); and (5) an abbreviated cognitive battery targeting domains of verbal/ visual memory (short-form California Verbal Learning Test, 2nd edition [CVLT-II; 6], Rey Complex Figure Test [RCFT; 7]), attention, processing speed, working memory (Brief Assessment of Cognition in Schizophrenia [BACS; 8], selected subtests), executive functioning (Victoria Stroop Test; [9], Trail Making Test; [10]) and social cognition (Reading the Mind in the Eyes task; [11]).

Data were collected by trained research personnel, with cognitive measures interpreted by a Clinical Neuropsychologist (GM). Where test-re-test normative data was available, variability over serial assessments was analysed via reliable change indices (RCI), using a Microsoft Excel template developed by Duff [12]. Standard RCI formula [13] were applied to symptom-based measures, with cognitive data analysed using formula, accounting for practice effects, test reliability and regression to the mean [14]. Values of +/- 1.645 were used to infer statistically significant change at the 10% level. Cognitive test data were also considered with reference to age-based normative data as per standard clinical neuropsychology practice [10].

S1b: Procedures for Anti-Neuronal Antibody Testing

Anti-NMDAR IgG antibodies were measured in diluted serum (1:10) or CSF (neat) as per manufacturer's instructions using a commercial assay containing primate hippocampus, primate cerebellum, NMDAR transfected Human Embryonic Kidney (HEK) 293 cells and non-transfected control HEK293 cells (Euroimmun, Germany). Paraneoplastic neuronal antibodies (ANNA-1/2, PCA1/2, Ma/Ta, CV2 and SOX-1) and glial fibrillary acidic protein (GFAP) were measured in diluted serum (1:40) or neat CSF, by indirect immunofluorescence on commercial primate cerebellum and cerebrum and mouse stomach substrate (INOVA, USA). For voltage-gated potassium channel (VGKC) antibodies, sera were tested by radio-immunoassay (RSR, England), with positive samples further assessed by indirect immunofluorescence on biochips of leucine-rich glioma-inactived-1 (LG1) and contactin-associated protein-like-2 (CASPR-2) transfected HEK293 cells (Euroimmun, Germany). Anti-alpha-amino-3-hydroxy-5-methyl-4-isoxazolepropionic acid (AMPA) and gamma-aminobutyric acid (GABAb) receptor IgG antibodies were measured in diluted serum (1:20) or neat CSF using a commercial assay containing AMPA-1, AMPA2 or GABAb transfected HEK293 cells (Euroimmun, Germany). Glutamic acid decarboxylase (GAD) antibodies were measured using indirect immunofluorescence testing on commercial primate cerebellum, cerebrum and rodent stomach sections and a commercial ELISA (RSR, UK).

References

1. Kay, S.R., L.A. Opler, and A. Fiszbein, *Positive and Negative Syndrome Scale (PANSS) rating manual*. 1986, Bronx, NY: Department of Psychiatry, Albert Einstein College of Medicine/ Mentefiore Medical Centre, and Schizophrenia Research Unit, Bronx Psychiatric Centre.
2. Lovibond, S.H. and P.F. Lovibond, *Manual for the Depression Anxiety Stress Scales (2nd Ed)*. 1995, Sydney, NSW: Psychology Foundation.
3. Patel, N., et al., *Simple and reliable determination of the modified rankin scale score in neurosurgical and neurological patients: the mRS-9Q.* Neurosurgery, 2012. **71**(5): p. 971-5; discussion 975.
4. American Psychiatric Association, *Diagnostic and Statistical Manual of Mental Disorders, 4th ed*. 2000, Washington, DC: American Psychiatric Association.
5. Hawthorn, G., J. Richardson, and R. Osborne, *The Assessment of Quality of Life (AQoL) instrument: a psychometric measure of health-related quality of life.* Quality of Life Research, 1999. **8**: p. 209-224.
6. Delis, D.C., *California verbal learning test manual, 2nd edition*. 2000, San Antonio, Texas: Psychological Corporation.
7. Osterrieth, P.A., *Le test de copie d'une figure complexe; contribution à l'étude de la perception et de la mémoire. Test of copying a complex figure; contribution to the study of perception and memory.* Archives of Psychology, 1944. **30**: p. 206-356.
8. Keefe, R.S.E., et al., *The Brief Assessment of Cognition in Schizophrenia: reliability, sensitivity, and comparison with a standard neurocognitive battery.* Schizophrenia Research, 2004. **68**(2-3): p. 283-297.
9. Spreen, O. and E. Strauss, *A compendium of neuropsychological tests, 2nd edition*. 1998, New York: Oxford University Press.
10. Sherman, E.M.S., J.E. Tan, and M. Hrabok, *A compendium of neuropsychological tests: fundamentals of neuropsychological assessment and test reviews for clinical practice, 4th edition*. Vol. New York. 2022: Oxford University Press.
11. Baron-Cohen, S., et al., *The "Reading the Mind in the Eyes" Test Revised Version: A Study with Normal Adults, and Adults with Asperger Syndrome or High-functioning Autism.* Journal of Child Psychology and Psychiatry, 2003. **42**(2): p. 241-251.
12. Duff, K., *Evidence-based indicators of neuropsychological change in the individual patient: relevant concepts and methods.* Arch Clin Neuropsychol, 2012. **27**(3): p. 248-61.
13. Jacobson, N.S. and P. Truax, *Clinical significance: a statistical approach to defining meaningful change in psychotherapy research.* Journal of Consulting and Clinical Psychology, 1991. **59**: p. 12-19.
14. Maassen, G.H., E.R. Bossema, and N. Brand, *Reliable change assessment with practice effects in sport concussion research: a comment on Hinton-Bayre.* Br J Sports Med, 2006. **40**(10): p. 829-33.

Supplementary Material 2

S2a: Comparison of group demographics, risk group classification, and symptom severity, in those who completed serological testing vs. those who declined.

Variables	*Blood Test Status*		Test Statistic	Effect Size
	Completed	Refused		
Age – M (SD)	34.0 (12.1)	35.6 (11.2)	$t(886) = -1.38, p = .168$	-
CGI – M (SD)	5.0 (1.5)	4.1 (1.4)	$t(884) = 6.39, p <.001$	Cohen's $d = .60$

	Blood Test Status						Total
	Completed			Refused			
Sex	*n*	%	Standardised Residual	*n*	%	Standardised Residual	
Male	439	58.2	-0.4	88	65.2	0.9	527
Female	315	41.8	0.5	47	34.8	-1.1	362
**Total**	754			135			889

$X^2(1, N = 889) = 2.29, p = .153$

	Blood Test Status						Total
	Completed			Refused			
Group	*n*	%	Standardised Residual	*n*	%	Standardised Residual	
High Risk EP	122	93.8	1.1	8	6.2	-2.6	130
Low Risk EP	230	94.7	1.6	13	5.3	-3.9	243
High Risk PP	290	78.2	-1.4	81	21.8	3.3	371
Low Risk PP	112	77.8	-0.9	32	22.2	2.2	144
**Total**	754			134			888

$X^2(3, N = 888) = 44.97, p < .001$; Cramer's $V = .225, p < .001$

S2b: Comparison of group demographics and symptom severity, in those who completed serological testing

Variables	*Group*		Test Statistic	Effect Size
	EP	PP		
Age – M (SD)	28.5 (10.9)	38.9 (11.0)	$t(752) = -12.86, p < .001$	Cohen's $d = -.94$
CGI – M (SD)	5.4 (1.2)	4.7 (1.7)	$t(751) = 6.99, p <.001$	Cohen's $d = .51$

	Sex						Total
	Male			Female			
Duration Group	*n*	%	Standardised Residual	*n*	%	Standardised Residual	
EP	194	55.1	-0.8	158	44.9	0.9	352
PP	245	60.9	0.7	157	39.1	-0.8	402
**Total**	439			315			754

$X^2(1, N =754) = 2.62, p = .105$

	Sex						Total
	**Male**			**Female**			
**Sx Duration**	***n***	**%**	**Standardised Residual**	***n***	**%**	**Standardised Residual**	
High Risk EP	57	46.7	-1.7	65	53.3	2.0	122
Low Risk EP	137	59.6	0.3	93	40.4	-0.3	230
High Risk PP	180	62.1	0.9	110	37.9	-1.0	290
Low Risk PP	65	58.0	0.0	47	42.0	0.0	112
**Total**	439			315			754

$X^2(3, N = 754) = 8.57, p < .035$; Cramer's $V = .107, p < .035$

S2c: Blood test result x risk group analysis

	Blood Test Result						Total
	**Negative**			**Positive**			
**Group**	***n***	**%**	**Standardised Residual**	***n***	**%**	**Standardised Residual**	
High Risk EP	114	93.4	-0.5	8	6.6	3.0	122
Low Risk EP	225	97.8	0.0	5	2.2	-0.2	230
High Risk PP	286	98.6	0.2	4	1.4	-1.1	290
Low Risk PP	111	99.1	0.2	1	0.9	-1.0	112
**Total**	736			18			754

$X^2(3, N = 754) = 11.49, p < .009$; Cramer's V = .123, $p < .009$;
Two cells (25%) had expected counts of <5 so Fisher's exact test was applied;
Fisher-Freeman-Halton Exact Test = 8.77, $p < .021$

Supplementary Material 3

S3a. Baseline and 12-month symptom, functional, and cognitive assessment results: consenting seropositive cases, early psychosis.

Case #	One	Two	Three	Four	Five	Six	Seven
**Psychotic Symptoms (PANSS[1]; Initial, 12 months)**	P7, N10, G23, T40<br>(Normal/ Borderline)	P10, N13, G28, T51<br>(Borderline/ Mild)	P11, N18, G33, T62<br>(Mild)	P11, N10, G24, T45<br>(Borderline)	P22, N23, G45, T90<br>(Markedly Ill)	P13, N19, G36, T68<br>(Mild/ Moderate)	P12, N10, G28, T50<br>(Borderline/ Mild)
	P7, N11, G27, T45<br>(Borderline)<br>50% ↑	-	P8, N8, G19, T35<br>(Normal/ Borderline)<br>84% ↓	P8, N10, G27, T45<br>(Borderline)<br>0% change	P21, N16, G33, T70<br>(Mild/ Moderate)<br>33% ↓	P21, N18, G42, T81<br>(Moderately/ Markedly Ill)<br>34% ↑	-
**Psychological Distress (DASS; Initial, 12 months)**	D – Normal<br>A – Normal<br>S - Normal	D – Mild<br>A – Normal<br>S - Normal	D – Ext Severe<br>A – Ext Severe<br>S – Severe	D – Normal<br>A – Mild<br>S – Mild	D – Mild<br>A – Moderate<br>S – Normal	D – Severe<br>A – Moderate<br>S – Normal	D – Normal<br>A – Normal<br>S – Normal
	D – Normal<br>A – Normal<br>S – Normal<br>(Stable)	-	D- Normal<br>A – Normal<br>S – Normal<br>(Improvement)	-	D – Mild<br>A – Normal<br>S – Normal<br>(Improvement)	D – Mild<br>A – Mild<br>S – Normal<br>(Improvement)	-
**Disability (MRS; Initial, 12 months)**	2 (slight disability)	2 (slight disability)	2 (slight disability)	1 (no disability)	3 (moderate disability)	3 (moderate disability)	2 (slight disability)
	2 (slight disability)	-	0 (no symptoms)	1 (no disability)	3 (moderate disability)	3 (moderate disability)	-
**Social & Occupational Functioning (SOFAS; Initial, 12 months)**	67 (some difficulty)	65 (some difficulty)	60 (moderate difficulty)	80 (slight difficulty)	43 (serious impairment)	51 (moderate difficulty)	65 (some difficulty)
	78 (slight difficulty)	-	81 (good function)	78 (slight difficulty)	46 (serious impairment)	45 (serious impairment)	-
**Quality of Life (AQoL; Initial, 12 months)**	81	89	69	94	56	78	97
	72	-	94	94	83	92	-

COGNITIVE DATA														
**Verbal memory**	Time 1	Time 2	Time 1	Time 2	Time 1	Time 2	Time 1	Time 2	Time 1	Time 2	Time 1	Time 2	Time 1	Time 2
Age-Based Descriptor	Normal	Normal	Ext Low	-	Normal	Normal	Normal	Normal	Ext Low	Borderline	Borderline	Borderline	Normal	-
Change	Stable		-		Stable		Stable		Stable		Stable		-	
**Visual memory**														
Age-Based Descriptor	Normal	Normal	Ext Low	-	Normal	Normal	Normal	Normal	Ext Low	Ext Low	Ext Low	Ext Low	Ext Low	-
Change	Declined		-		Stable		Stable		Stable		Stable		-	
**Visual search, speed**														
Age-Based Descriptor	Normal	Normal	Normal	-	Normal	Normal	Ext Low	Normal	Ext Low	Ext Low	Ext Low	Borderline	Normal	-
Change	Stable		-		Stable		Improved		Decline		Stable		-	
**Working memory**														
Age-Based Descriptor	Normal	Normal	Normal	-	Normal	Normal	Borderline	Normal	Ext Low	Borderline	Ext Low	Ext Low	Normal	-

[1] Rescaled to 0 prior to percentage change calculation, as per: Leucht (2014). Measurements of response, remission, and recovery in schizophrenia and examples of their clinical application, *J Clin Psychiatry*, 75 (Suppl 1): 8 – 14.

**Case #**	**One**		**Two**		**Three**		**Four**		**Five**		**Six**		**Seven**	
Change	Stable		-		Stable		Stable		Stable		Stable		-	
**Verbal fluency**														
Age-Based Descriptor	Normal	Normal	Ext Low	-	Normal	Normal	Normal	Normal	Ext Low	Ext Low	Ext Low	Ext Low	Normal	-
Change	Improved		-		Improved		Improved		Improved		Stable		-	
**Mental flexibility**														
Age-Based Descriptor	Normal	Normal	Normal	-	Borderline	Ext Low	Normal	Normal	Ext Low	Ext Low	Ext Low	Ext Low	Normal	-
Change	Stable		-		Declined		Stable		Improved		Decline		-	
**Response inhibition**														
Age-Based Descriptor	Normal	Normal	Ext Low	-	Normal	Normal	Ext Low	Normal	Ext Low	Ext Low	Borderline	Ext Low	Normal	-
Change	Stable		-		Stable		Stable		Stable		Stable		-	
**Social cognition**														
Age-Based Descriptor	Normal	Normal	Normal	-	Normal	Normal	Normal	Normal	Borderline	Borderline	Ext Low	Ext Low	Normal	-
Change	Stable		-		Improved		Stable		Stable		Decline		-	

Abbreviations. A = Anxiety Scale; AQoL = Assessment of Quality of Life; D = Depression Scale; DASS = Depression Anxiety Stress Scale; Ext = Extremely; G = General Psychopathology Scale; MRS = Modified Rankin Scale; N = Negative Symptoms Scale; P = Positive Symptoms Scale; PANSS = Positive and Negative Syndrome Scale; S = Stress Scale; SOFAS = Social and Occupational Functioning Assessment Scale; T = Total Score.

S3b. Early psychosis seropositive longitudinal cohort summary.

Five seropositive EP patients were followed up at baseline and 12 months (Cases 1, 3 – 6). In Case 1 where NMDAr encephalitis was confirmed and treated, neurological progress was slow but outcomes appeared favourable. Two were NMDAr seropositive, with lumbar puncture not performed (Case 3, 5). Both were managed with psychiatric care alone, with meaningful improvements noted in psychotic symptoms, and self-reported psychological distress. Case 5 remained functionally impaired in the setting of lifelong neurodevelopmental diagnoses, but reported improved quality of life and was no longer anti-neuronal Ab seropositive at follow-up. Case 3 demonstrated functional recovery and improved quality of life. He was seropositive at both time-points. Case 4 was VGKC positive, LGi1/Caspr-2 negative, diagnosed with drug-induced psychosis managed with psychiatric care alone. All assessments were largely unchanged. Anti-neuronal Ab were not detected at follow-up. Case 6 was Caspr-2 seropositive, managed solely with psychiatric care supported by neurological consultation. Psychotic symptoms did not respond to psychiatric treatment, and cognitive decline was evident in the setting of substance-use. Subjective quality of life and psychological distress had improved by follow-up. Functioning remained impaired.

S3c. Baseline and 12-month symptom, functional, and cognitive assessment results: consenting seropositive cases, persistent psychosis.

Case #	Eight		Nine		Ten	
**Psychotic Symptoms (PANSS[2]; Initial, 12 months)**	P16, N19, G34, T69 (Mild/ Moderate)		P7, N10, G23, T40 (Normal/ Borderline)		P7, N13, G24, T44 (Borderline)	
	P14, N14, G31, T59 (Mild), 25% ↓		P10, N10, G23, T43 (Borderline), 30% ↑		P7, N13, G18, T38 (Normal/ Borderline), 43% ↓	
**Psychological Distress (DASS; Initial, 12 months)**	D – Mild<br>A – Normal<br>S – Normal		D – Normal<br>A – Normal<br>S – Normal		D – Normal<br>A – Normal<br>S – Normal	
	D – Normal<br>A – Normal<br>S – Normal<br>(Stable)		D – Normal<br>A – Normal<br>S – Normal<br>(Stable)		D – Normal<br>A – Normal<br>S – Normal<br>(Stable)	
**Disability (MRS; Initial, 12 months)**	3 (moderate disability)		3 (moderate disability)		3 (moderate disability)	
	3 (moderate disability)		3 (moderate disability)		1 (no disability)	
**Social & Occupational Functioning (SOFAS; Initial, 12 months)**	51 (moderate difficulty)		59 (moderate difficulty)		61 (some difficulty)	
	52 (moderate difficulty)		59 (moderate difficulty)		70 (some difficulty)	
**Quality of Life (AQoL; Initial, 12 months)**	72		78		92	
	89		86		89	
**COGNITIVE DATA**						
**Verbal memory**	**Time 1**	**Time 2**	**Time 1**	**Time 2**	**Time 1**	**Time 2**
Age-Based Descriptor	Normal	Normal	Borderline	Ext Low	Borderline	Normal
Change	Stable		Stable		Stable	
**Visual memory**						
Age-Based Descriptor	Normal	Normal	Normal	Normal	Ext Low	Normal
Change	Stable		Stable		Improved	
**Visual search, speed**						
Age-Based Descriptor	Normal	Normal	Normal	Normal	Normal	Normal
Change	Stable		Stable		Stable	
**Working memory**						
Age-Based Descriptor	Normal	Borderline	Normal	Normal	Ext Low	Ext Low
Change	Stable		Stable		Stable	
**Verbal fluency**						
Age-Based Descriptor	Normal	Normal	Normal	Normal	Normal	Normal
Change	Stable		Stable		Stable	
**Mental flexibility**						

[2] Rescaled to 0 prior to percentage change calculation, as per: Leucht (2014). Measurements of response, remission, and recovery in schizophrenia and examples of their clinical application, *J Clin Psychiatry*, 75 (Suppl 1): 8 – 14.

Age-Based Descriptor	Ext Low	Borderline	Normal	Normal	Normal	Ext Low
Change	Stable		Stable		Declined	
**Response inhibition**						
Age-Based Descriptor	Normal	Normal	Normal	Normal	Normal	Normal
Change	Stable		Stable		Stable	
**Social cognition**						
Age-Based Descriptor	Normal	Borderline	Normal	Ext Low	Borderline	Normal
Change	Stable		Stable		Stable	

Abbreviations. A = Anxiety Scale; AQoL = Assessment of Quality of Life; D = Depression Scale; DASS = Depression Anxiety Stress Scale; Ext = Extremely; G = General Psychopathology Scale; MRS = Modified Rankin Scale; N = Negative Symptoms Scale; P = Positive Symptoms Scale; PANSS = Positive and Negative Syndrome Scale; S = Stress Scale; SOFAS = Social and Occupational Functioning Assessment Scale; T = Total Score.

S3d. Persistent psychosis seropositive longitudinal cohort summary.

One case of autoimmune psychosis was confirmed and treated from the PP cohort, with notable improvements in psychotic symptoms identified. Two additional high risk PP cases were followed-up. Case 9 was NMDAr seropositive, CSF negative, managed with psychiatric care alone for paranoid schizophrenia. Psychotic symptoms increased by 30% to the borderline illness range. Psychological distress ratings were normal, unchanged over time. Cognitive functioning was largely normal, with some variability. Functional deficits were noted on both occasions. A slight increase in health-related quality of life was noted. Case 10 was diagnosed with schizoaffective disorder and was GFAP seropositive at both timepoints. CSF was not able to be collected. Psychotic symptoms improved with psychiatric care alone, psychological distress ratings were normal at both time-points, and health-related quality of life was largely unchanged. He presented with social and occupational functional difficulties at both time-points, but neurological disability had resolved.
